# Hierarchical Carbon Microtube@Nanotube Core–Shell Structure for High-Performance Oxygen Electrocatalysis and Zn–Air Battery

**DOI:** 10.1007/s40820-020-00435-z

**Published:** 2020-04-20

**Authors:** Wenfu Xie, Jianming Li, Yuke Song, Shijin Li, Jianbo Li, Mingfei Shao

**Affiliations:** 1 State Key Laboratory of Chemical Resource Engineering, Beijing University of Chemical Technology, Beijing, 100029 People’s Republic of China; 2 Petroleum Geology Research and Laboratory Center, Research Institute of Petroleum Exploration and Development (RIPED), PetroChina, Beijing, 100083 People’s Republic of China

**Keywords:** Hierarchical structure, Carbon microtube@nanotube, Core–shell, Zinc–air battery

## Abstract

Zinc–air batteries (ZABs) hold tremendous promise for clean and efficient energy storage with the merits of high theoretical energy density and environmental friendliness. However, the performance of practical ZABs is still unsatisfactory because of the inevitably decreased activity of electrocatalysts when assembly into a thick electrode with high mass loading. Herein, we report a hierarchical electrocatalyst based on carbon microtube@nanotube core–shell nanostructure (CMT@CNT), which demonstrates superior electrocatalytic activity for oxygen reduction reaction and oxygen evolution reaction with a small potential gap of 0.678 V. Remarkably, when being employed as air–cathode in ZAB, the CMT@CNT presents an excellent performance with a high power density (160.6 mW cm^−2^), specific capacity (781.7 mAhg_Zn_^−1^) as well as long cycle stability (117 h, 351 cycles). Moreover, the ZAB performance of CMT@CNT is maintained well even under high mass loading (3 mg cm^−2^, three times as much as traditional usage), which could afford high power density and energy density for advanced electronic equipment. We believe that this work is promising for the rational design of hierarchical structured electrocatalysts for advanced metal-air batteries.

**Electronic supplementary material:**

The online version of this article (10.1007/s40820-020-00435-z) contains supplementary material, which is available to authorized users.

## Highlights

- Hierarchical carbon microtube@nanotube (CMT@CNT) core–shell nanostructure is successfully synthesized.
- The CMT@CNT shows superior electrocatalytic activity for oxygen reduction reaction and oxygen evolution reaction.
- A mass-loading independent high performance for zinc–air battery is achieved on the CMT@CNT.

**Electronic supplementary material:**

The online version of this article (10.1007/s40820-020-00435-z) contains supplementary material, which is available to authorized users.

## Graphical abstract

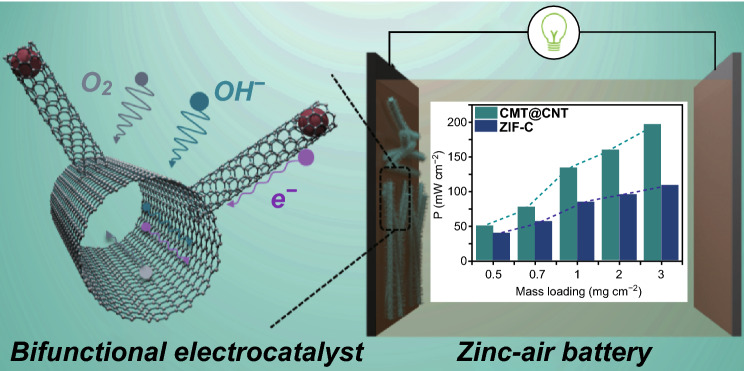

## Introduction

Zinc-air batteries (ZABs) have been targeted as a promising clean power candidate with the merits of high theoretical energy density, environmental friendliness, and low cost [1, 2]. The overall performance of rechargeable ZABs is critically dependent on the electrode with superior bifunctional behavior for both oxygen reduction reaction (ORR) and oxygen evolution reaction (OER) [3]. Noble metal-based materials, such as Pt- and Ir-based, have long been regarded as the state-of-the-art electrocatalysts for ORR and OER, respectively, but their large-scale application has been impeded by scarcity, high cost, and insufficient stability [4]. Recently, transition metal-nitrogen-doped carbon (M–N–C) has emerged as one of the most promising candidates for electrocatalyzing both ORR and OER due to their abundant precursors, high activity, and selectivity [5, 6]. Up to date, various M–N–C (i.e., Co–N–C, Fe–N–C, and Ni–N–C) have been thoroughly explored from the perspectives of material synthesis, structure design, and activity regulation [7–13]. Although these progresses, the performance of M–N–C electrocatalysts in practical ZABs is still unsatisfactory because the theoretical activity will seriously decrease when assembly the active materials to functional electrodes, which probably caused by the limited electrolyte diffusion and the repressed utilization of active sites in the compacted film [14]. This drawback is especially serious when the electrocatalyst is employed under high mass loading because just exposed sites can participate in the catalytic reactions.

To maintain the activity and stability of the electrocatalysts on the electrode, an unobstructed electrolyte diffusion process and easily exposed active sites are essential [15]. One effective approach to meet this requirement is engineering electrocatalysts with rational and elaborate nanostructures. A great deal of nanostructured electrocatalysts has been exploited and applied, including nanosheets [16, 17], nanowires [18, 19], nanotubes [20, 21], microspheres [22, 23], yolk-shelled [24, 25] and mutilshelled materials [26, 27], and so on, which exhibit much enhanced performance toward various electrochemical reactions. Despite these research progresses, there is still a concern over the declined overall electrochemical performance in practical energy applications, especially under high mass loading [28]. To overcome this issue, the construction of hierarchical structure could realize stable output of electrochemical performance even under high mass loading, which is promising for high-performance energy devices [29]. The hierarchical structure will reduce embedding of nanomaterials and provide a large specific surface area and rich porosities in practical environment [30–32]. Besides, the hierarchical structure will also facilitate the infiltration and transport of gas molecules and liquid electrolyte during electrochemical reactions [33, 34]. Unfortunately, it is still a challenge how to assemble low-dimensional nanomaterials on the meso- or micro-structure to achieve a hierarchical electrocatalyst with high performance when using as a thick functional electrode.

Herein, we successfully demonstrate a hierarchical carbon microtube@nanotube core–shell nanostructure (denoted as CMT@CNT) as highly active and stable electrocatalysts for both ORR and OER to achieve high performance rechargeable ZAB. The hierarchical CMT@CNT with dense nanotube vertically rooted on the hollow microtube was synthesized through in situ growth of metal organic frameworks and the subsequent pyrolysis treatment. Moreover, the structure–activity relationship has been rationally discussed through various characterizations. The as-obtained CMT@CNT exhibits a positive half-wave potential of 0.88 *V*_RHE_ (versus reversible hydrogen electrode, RHE) for ORR, a low overpotential of 328 mV for OER and a small potential gap of 0.678 V, which exceeds that of most reported carbon-based electrocatalysts. The ZAB assembled by this CMT@CNT demonstrates a high power density of 160.6 mW cm^−2^ and specific capacity of 781.7 mAhg_Zn_^−1^ as well as long cycle stability over 117 h (351 cycles) at a current density of 20 mA cm^−2^. Moreover, CMT@CNT maintains its ZAB performance well even under high mass loading (3 mg cm^−2^, 3 times as much as traditional usage), which should meet the high demand of high power density and energy density for advanced electronic equipment.

## Experimental Section

### Synthesis of Polypropylene@polydopamine(PP@PDA)

The dopamine was firstly polymerized and coated on the surface of polypropylene (PP) nanowire by a reported method [35]. Briefly, 0.3 g PP wetting by ethanol and 160 mg dopamine were immersed into a 400 mL deionized water. Then, 5 mL tris–HCl was dropped in above solution slowly. The solution was stirred for 24 h at room temperature. After that, polydopamine (PDA)-coated PP (denoted as PP@PDA) was withdrawn and washed with water several times and then put into an oven to dry at 60 °C overnight.

### Synthesis of PP@PDA@ZIF-67

Typically, 0.1 g PP@PDA nanowire was immersed into a 100 mL methanol solution containing of 0.05 M Co(NO_3_)_2_ 6H_2_O. Then, another 100 mL methanol solution containing of 0.4 M 2-methylimidazole was rapidly poured into above solution under stirring for 30 min. After that, the products (denoted as PP@PDA@ZIF-67) was collected by centrifugation and washed with methanol several times and then put into an oven to dry at 60 °C overnight.

### Synthesis of CMT@CNT

The PP@PDA@ZIF-67 was placed in furnace and subjected with a pyrolysis treatment at 800 °C for 3 h under N_2_/H_2_ (9/1) atmosphere. The heating rate is 5 °C min^−1^. The obtained CMT@CNT was directly used and without any further treatment.

For comparison, CMT was synthesized via directly pyrolyzing PP@PDA nanowire with the same procedure of CMT@CNT. ZIF-67 nanoparticle was also synthesized with the same procedure of PP@PDA@ZIF-67 except the use of PP@PDA. Then, ZIF-67 nanoparticle was subjected with the same pyrolysis treatment of CMT@CNT to obtain the ZIF-C nanoparticle.

### Characterizations

The morphology of the synthesized electrocatalysts was characterized by scanning electron microscopy (SEM; Zeiss SUPRA 55) and transmission electron microscopy (TEM; Philips Tecnai 20) and high-resolution TEM (HRTEM; JEOL JEM-2010). The structure, chemical composition and physical properties were characterized by X-ray diffraction patterns (XRD; Shimadzu XRD-6000 diffractometer), Raman microspectrometer (Renishaw, inVia-Reflex, 532 nm), X-ray photoelectron spectra (XPS; Thermo VG ESCALAB 250) and N_2_ sorption isotherm (Quantachrome Autosorb-1CVP analyzer).C K-edge and N K-edge XANES spectra were measured at the beamline 1W1B of the Beijing Synchrotron Radiation Facility (BSRF, Beijing), China.

### Electrochemical Measurements

Electrochemical measurements were carried out by a CHI760E electrochemical workstation (Shanghai Chenhua Instrument Co., China) with a conventional three-electrode system. A saturated calomel electrode (SCE) (3.5 M KCl) and carbon rod electrode were served as the reference and counter electrode, respectively. A rotating disk electrode (RDE, 5 mm in diameter) and rotating ring-disk electrode (RRDE, 4 mm in diameter and the area of Pt ring is 0.224 cm^2^) were served as working electrode. 4 mg of the as-obtained electrocatalysts was dispersed in1 mL mixture of water, isopropanol and Nafion (5 wt%) with the ratio of 770:200:30. Then, the above electrocatalyst ink was sonicated for at least 30 min to form a homogeneous suspension. After that, the electrocatalyst ink was dropped carefully on the working electrode with a loading of 0.2 mg cm^‒2^. For ORR measurements, all curves were obtained in O_2_-saturated 0.1 M KOH electrolyte solution (pH = 13). The cyclic voltammetry (CV) curves were conducted with a scan rate of 100 mV s^−1^ from 1.2 to 0.2 V versus RHE. LSV curves were measured at various rotation rates (400, 625, 900, 1225, 1600, and 2025 rpm) with a scan rate of 5 mV s^−1^ from 1.2 to 0.1 V versus RHE. LSV curves in RRDE measurements were measured at 1600 rpm with the same potential range of RDE measurements. The constant E of ring electrode is 0.5 V. The stability test was carried out by the *i*–*t* curve at 1600 rpm. The CV curves for calculating the ECSA were measured with the scan rates of 20, 40, 60, 80, 100, and 120 mV s^‒1^ from 0.508 to 0.608 versus RHE. The EIS measurements were performed at onset potential in the frequency range of 0.1–10^5^ Hz with an amplitude of 5 mV. For OER measurements, all curves were obtained in 1 M KOH electrolyte solution (pH = 14). LSV curves were measured with a scan rate of 5 mV s^−1^ from 1.1 to 1.8 V versus RHE (90% iR compensation). Multi-potential step curves were measured at overpotential of 300, 350, 400, 450, 500 mV and each applied potential was maintained for 600 s.

The electron transfer number from RDE measurements was calculated by Koutecky–Levich equation (Eqs. 1–3):

1 $$\frac{1}{j} = \frac{1}{{j_{\text{L}} }} + \frac{1}{{j_{\text{K}} }} = \frac{1}{{B\omega^{1/2} }} + \frac{1}{{j_{\text{K}} }}$$

2 $$B = 0.62nFC_{0} \left( {D_{0} } \right)^{2/3} v^{ - 1/6}$$

3 $$j_{K} = nFkC_{0}$$

where *j* is the measured current density; *j*_K_ and *j*_L_ are the kinetic- and diffusion-limiting current density; *ω* is the RDE rotating rate; *F* is the Faraday constant (*F *= 96,485 C mol^−1^); *C*_0_ is the bulk concentration of O_2_ (1.2 × 10^−6^ mol cm^−3^); *D*_0_ is the diffusion coefficient of O_2_ in 0.1 M KOH solution (1.9 × 10^−5^ cm^2^ s^−1^); *ν* is the kinematic viscosity of the electrolyte (0.01 cm^2^ s^−1^), and *k* is the electron transfer rate constant.

The H_2_O_2_ yield and electron transfer number from RRDE measurements were calculated by Eqs. (4) and (5):

4 $$n = 4 \times \frac{{i_{\text{d}} }}{{i_{\text{r}} /N + i_{\text{d}} }}$$

5 $${\text{HO}}_{2}^{ - } \left( {\text{\%}} \right) = 200 \times \frac{{i_{\text{n}} /N}}{{i_{\text{r}} /N + i_{\text{d}} }}$$

where *i*_d_ and *i*_r_ are disk current and ring current, respectively, and *N *= 0.37 is the current collection efficiency of the Pt ring.

The reversible hydrogen electrode (RHE) was converted from saturated calomel reference electrode (SCE) by Nernst equation (Eq. 6):

6 $$E_{\text{RHE}} = E_{\text{SCE}} + 0.0591pH + E_{\text{SCE}}^{0}$$

where $E_{\text{SCE}}^{0}$ is the standard potential of SCE at 25 °C (0.24 V).

### For Zinc–Air Battery Measurements

Zinc–air battery (ZAB) was measured in a home-built electrochemical cell, where electrocatalyst supported on carbon cloth (mass loading of 1 mg cm^−2^) as air cathode and Zn foil as the anode with CHI760E electrochemical working station. 6 M KOH + 0.2 M zinc acetate was used as the electrolyte. The polarization curves were recorded with a scan rate of 5 mV s^−1^ from 1.6 to 0 V. The galvanostatic discharge–charge cycling curves were recorded 20 min per cycle at a current density of 20 mA cm^−2^. The galvanostatic discharge curves were recorded at a current density of 10 mA cm^−2^. For all-solid-state ZAB, home-made alkaline polyvinyl alcohol (PVA) gel was used as solid electrolyte. The synthesis of PVA gel was obtained from the previous works. Both the current density and power density were normalized to the effective surface area of air electrode.

The specific capacity was calculated according to Eq. (7):

7 $${\text{Specific}}\;{\text{capacity}} = \frac{{{\text{current}}*{\text{running}}\;{\text{time}}}}{{{\text{weight}}\;{\text{of}}\;{\text{consumed}}\;{\text{zinc}}}}$$

The energy density was calculated according to Eq. (8):

8 $${\text{Specific}}\;{\text{capacity}} = \frac{{{\text{current}}*{\text{running}}\;{\text{time}}*{\text{average}}\;{\text{discharge}}\;{\text{voltage}}}}{{{\text{weight}}\;{\text{of}}\;{\text{consumed}}\;{\text{zinc}}}}$$

## Results and Discussion

### Morphological and Structural Characterization

**Fig. 1:**
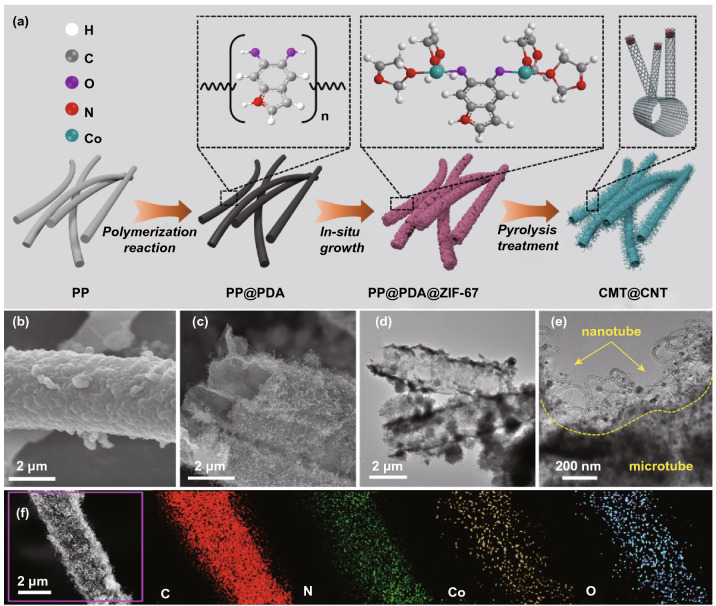
**a** Schematic illustration for the synthesis of CMT@CNT. SEM images of **b** PP@PDA@ZIF-67 and **c** CMT@CNT. **d**, **e** TEM and **f** SEM images and the corresponding elemental mapping of CMT@CNT

As schematically illustrated in Fig. 1a, the preparation of hierarchical CMT@CNT core–shell structure involves three steps: polymerization of dopamine on PP, in situ growth of zeolitic imidazolate framework (ZIF) and pyrolysis treatment. Briefly, PDA was firstly constructed on the surface of PP by dopamine polymerization (Figs. S1 and S2). Subsequently, ZIF-67 nanoparticles were assembled uniformly on PP@PDA directed by the strong interaction between amino in PDA and Co ions (denoted as PP@PDA@ZIF-67, Fig. 1b). Last, a pyrolysis treatment under N_2_/H_2_ (9/1) atmosphere was performed with a heating temperature of 800 °C and a heating time of 3 h to obtain CMT@CNT (Fig. 1c and S3). It is worth noting that the introduction of PDA nanolayer as confined layer could orderly assemble ZIF-67 nanoparticles on the surface of nanofiber and significantly alleviate the aggregation of ZIF-C particle during the pyrolysis process. TEM and HRTEM further confirm the successful formation of hierarchical microtube@nanotube core–shell nanostructure, where a large number of CNTs with a diameter of ~ 20 nm are uniformly rooted on the microtube (Figs. 1d, e, and S4). The nanoparticles embedded in CNTs show a lattice fringe of 0.204 nm, corresponding to the (111) plane of metallic Co. The Co precursor in ZIF-67 was reduced to metallic Co by surrounding carbonaceous species, which in situ catalyzing the formation of CNTs along with the increase in pyrolysis temperature [36]. Moreover, the energy-dispersive X-ray spectroscopy mapping result shows that C, N, Co, and O are uniformly distributed in the CMT@CNT (Fig. 1f). In contrast, polyhedral carbon particle with a small amount of CNT on the surface was synthesized through conducting ZIF-67 nanoparticle with the same pyrolysis parameters (denoted as ZIF-C, Fig. S5). In order to highlight the structure advantages of hierarchical microtube@nanotube core–shell nanostructure, carbon microtube (denoted as CMT) was also prepared via pyrolyzing PP@PDA (Fig. S6).

**Fig. 2:**
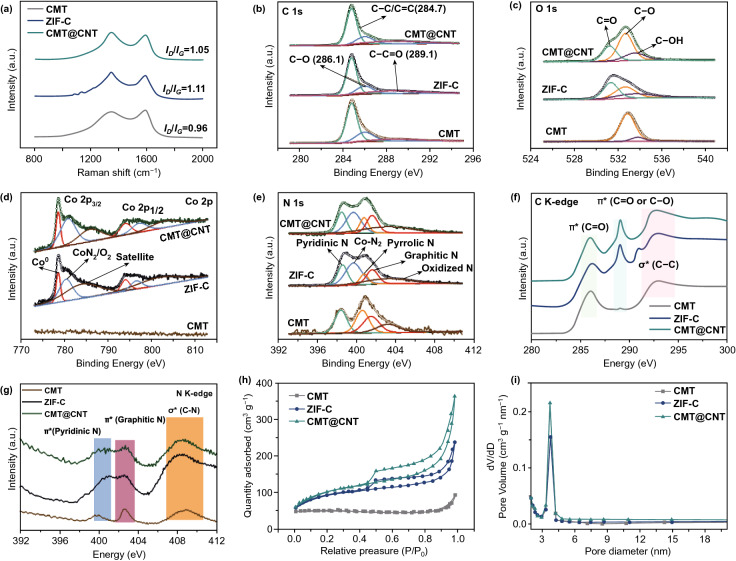
**a** Raman spectra, High-resolution XPS spectra of **b** C 1s, **c** O 1s, **d** Co 2p, and **e** N 1s, **f** C K-edge, and **g** N K-edge XANES, **h** N_2_ sorption isotherm, and **i** pore size distribution plots of CMT@CNT, ZIF-C, and CMT

The XRD patterns show that the resultant CMT@CNT, ZIF-C, and CMT share similar broad peak located between 20° and 30°, which is indexed to the (002) diffraction peak of graphitic carbon (Fig. S7). In addition, both CMT@CNT and ZIF-C show three peaks located at 44.2°, 51.5°, and 75.8°, matching well with the (111), (200), and (220) planes of face-centered cubic (*fcc*) Co phase [37]. Raman spectra depict that the relative intensity ratio between defect carbon (D band, 1350 cm^−1^) and graphitic carbon (G band, 1600 cm^−1^) of CMT@CNT is 1.05, smaller than that of ZIF-C (1.11), demonstrating the increased content of graphitic carbon caused by microtube skeleton, which could be beneficial for enhancing the electron transfer process (Fig. 2a) [38]. The element compositions and chemical states of CMT@CNT and references were further characterized by XPS and soft X-ray absorption spectroscopy (XAS). XPS spectra suggest that C, O, Co, and N element present in CMT@CNT and ZIF-C while Co element being absent in CMT (Fig. S8 and Table S1). The high intensity of C–C/C=C signal (284.7 eV) in the high-resolution C 1 s spectra of all samples indicate a typical graphitic carbon structure (Fig. 2b). The presence of O signal (C–O (286.1 eV) and O–C=O (289.1 eV)) can be caused by the adsorbed oxygen from the environmental atmosphere [39], which is confirmed by the high-resolution O 1 s spectra (Fig. 2c). The high-resolution Co 2p spectra of CMT@CNT and ZIF-C are deconvoluted into two pairs of metallic Co (778.6/794.0 eV) and CoN_*x*_/O_*x*_ (780.4/796.5 eV) in 2p_3/2_ and 2p_1/2_ peaks, respectively (Fig. 2d) [40]. The dopant of N is testified from the high-resolution N 1 s spectra, where five peaks of pyridinic-N (398.6 eV), Co-N_*x*_ (399.7 eV), pyrrolic-N (400.8 eV), graphitic-N (401.6 eV), and oxidized N (403.5 eV) are appeared in both CMT@CNT and ZIF-C (Fig. 2e) [41]. However, CMT@CNT shows a high content of graphitic-N (17.9%), larger than that of ZIF-C (15.3%), which is believed to contribute the enhanced electrocatalytic activity and electron transfer ability. The C K-edge soft X-ray absorption near edge structure (XANES) spectra of both three samples show two strong peaks at ~ 286.1 and ~ 292.8 eV in accordance with the C=C π* and C–C σ* excitation, meaning that high degree of graphitization (Fig. 2f). Another noticeable peak at ~ 289.1 eV in accordance with the C=O/C–O π* excitation is probably caused by the absorbed oxygen from atmosphere [42]. Three peaks at ~ 400.4, ~ 402.6, and 408.4 eV result from pyridinic-N π*, graphitic-N π*, and C-N σ* are appeared on CMT, ZIF-C, and CMT@CNT, respectively, which is in accordance with the result of XPS (Fig. 2g). N_2_ sorption isotherm results show that a high Brunauer–Emmett–Teller specific surface area of 354.27 m^2^ g^−1^ as well as a pore volume of 0.51 cm^3^ g^−1^ are achieved on CMT@CNT, which are larger than those of ZIF-C (320.57 m^2^ g^−1^, 0.29 cm^3^ g^−1^) and CMT (33.36 m^2^ g^−1^, 0.08 cm^3^ g^−1^) (Fig. 2h and Table S2). Both CMT@CNT and ZIF-C have a similar porous structure with an average pore size of ~ 8 nm (Fig. 2i). It is worth mentioned that the introduction of PDA can not only maintain the tubular structure and inhibit the agglomeration of ZIF particles during the high-temperature pyrolysis process, but also increase the content of N and graphitic N for CMT@CNT. Moreover, the unique hierarchical microtube@nanotube core–shell nanostructure with increased specific surface area will increase the available active sites and facilitate the electrolyte diffusion process.

### ORR and OER Properties

**Fig. 3:**
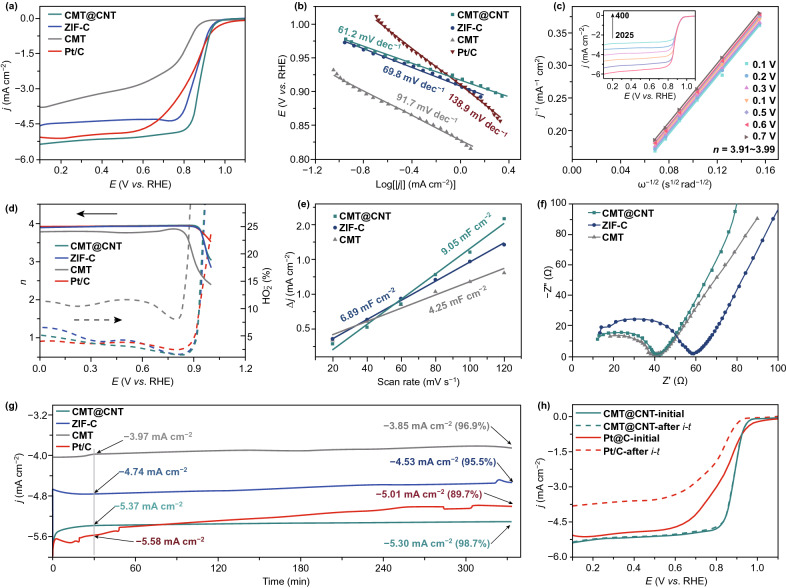
ORR performance of CMT@CNT, ZIF-C, CMT, and Pt/C. **a** LSV curves and **b** Tafel slopes. **c**Koutecky–Levich plots of CMT@CNT, inset: LSV curves at various rotation speeds. **d** Electron transfer number and H_2_O_2_ yield base on RRDE measurements. **e** ECSA and **f** Nyquist plots of CMT@CNT, ZIF-C, and CMT. **g ***I*-*t* curves, **h** LSV curves of initial and after *i*-*t* testing of CMT@CNT and Pt/C

The ORR electrocatalytic activities of CMT@CNT and comparative samples were firstly tested by a rotating disk electrode (RDE). Cyclic voltammetry (CV) curves show that obviously enhanced cathodic peak related to oxygen reduction for all electrocatalysts, and the most positive peak potential for CMT@CNT implies more superior ORR activity (Fig. S9). Moreover, CMT@CNT exhibits the highest ORR current density of − 5.47 mA cm^−2^ at 0.1 *V*_RHE_ and the most positive half-wave potential of 0.88 *V*_RHE_, indicating the highest ORR electrocatalytic activity among ZIF-C (− 4.61 mA cm^−2^, 0.86 *V*_RHE_), CMT (− 3.92 mA cm^−2^, 0.75 *V*_RHE_), and Pt/C (− 5.43 mA cm^−2^, 0.82 *V*_RHE_). The Tafel slope obtained from the LSV at 1600 rpm for CMT@CNT is 61.2 mV dec^−1^, which is smaller than that of ZIF-C (69.8 mV dec^−1^), CMT (91.7 mV dec^−1^), and Pt/C (138.9 mV dec^−1^), illustrating a better electron transfer process and more favorable ORR kinetics (Fig. 3b). It has been reported that the Co–N_*x*_ species of Co–N–C-based electrocatalyst are important active site for ORR [29]. As we can see from high-resolution Co 2p XPS spectra (Fig. 2d), there are abundant Co–N_*x*_ structure in CMT@CNT, which supposed to play an important role in electrocatalytic process. Besides Co–N_*x*_ species, the hierarchical structure of CMT@CNT endows large specific surface area and abundant pore structure, which dramatically promotes the exposure of active sites and improves the utilization rate of active sites. Furthermore, the electron transfer number of CMT@CNT obtained from LSV measurements at different rotation rates and the corresponding Koutecky–Levich plots is determined to be 3.95, highlighting a predominant 4-electron pathway (Figs. 3c and S10). Besides, the electron transfer number and H_2_O_2_ yield were monitored by the rotating ring-disk electrode (RRDE) measurements (Figs. 3d and S11, Table S3). The Pt-like properties of low H_2_O_2_ yield (3.29%) and electron transfer number (3.93) for CMT@CNT suggest the largely suppressed 2e^−^ reduction pathway and high selectivity for 4e^−^ reduction pathway. The highest electrochemical active surface (9.05 mF cm^−2^) is evidenced from the electrochemical double layer capacitance for CMT@CNT, indicating more active sites can be exposed and utilized in this structure (Figs. 3e and S12). The smallest diameter of the semicircle for CMT@CNT from the Nyquist plot demonstrates the lowest charge transfer resistance (Fig. 3f). After more than 330 min continuous testing, about 98.7% current retention was achieved on CMT@CNT, superior to Pt/C (89.7%), ZIF-C (95.5%), and CMT (96.9%). The LSV curve of CMT@CNT delivers negligible changes after *i*–*t* test, further demonstrating the durable long-term stability. Additionally, the morphology and structure of CMT@CNT after stability test were confirmed by SEM characterization, which nearly maintains the initial structure, demonstrating superior durability (Fig. S13). Moreover, the methanol poisoning experiments show that CMT@CNT remained almost constant activity with addition of methanol during *i*-*t* test, which is much superior to that of commercial Pt/C, indicating high tolerance against methanol crossover (Fig. S14).

**Fig. 4:**
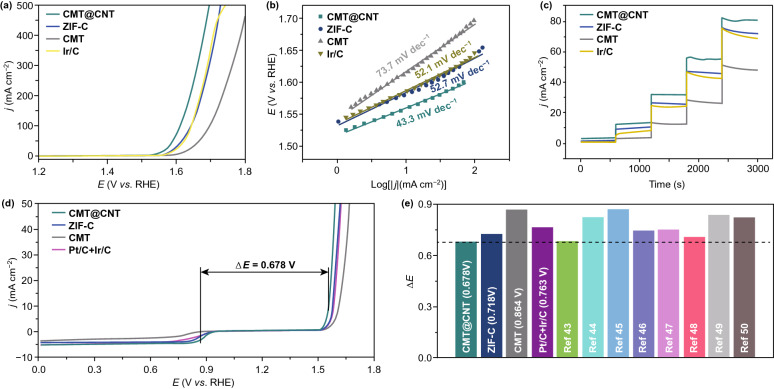
OER performance of CMT@CNT, ZIF-C, CMT, and Ir/C. **a** LSV curves, **b** Tafel slopes, and **c** multi-potential steps curves. **d** Overall LSV curves and **e**Δ*E* of CMT@CNT, ZIF-C, CMT, Pt/C + Ir/C, and the studies

Subsequently, the OER activity of these catalysts was further evaluated. LSV results show a largely reduced onset potential and overpotential and significantly boosted current density for CMT@CNT (Fig. 4a). Typically, CMT@CNT only requires an overpotential of 328 mV to drive a current density of 10 mA cm^−2^, less than that of ZIF-C (348 mV), CMT (384 mV), and Ir/C (353 mV), respectively. The excellent OER performance of CMT@CNT is further confirmed from the current density at large overpotential. For instance, at a potential of 1.65 *V*_RHE_, CMT@CNT exhibits a current density of 242.7 mA cm^−2^, which is ~ 2, ~ 8, and ~ 2 times larger than that of ZIF-C, CMT, and Ir/C, respectively. The enhanced OER activity of CMT@CNT is also evidenced from Tafel slope (43.3 mV dec^−1^), which is much smaller than that of ZIF-C (52.7 mV dec^−1^), CMT (73.7 mV dec^−1^), and Ir/C (52.1 mV dec^−1^), respectively, suggesting more favorable OER kinetics for CMT@CNT. Besides, CMT@CNT maintains similar performance with the initial value during continuous testing at overpotential of 300, 350, 400, 450, 500 mV, revealing a satisfactory durability toward OER. The potential gap (Δ*E*) between half-wave potential for ORR and overpotential for OER at a current density of 10 mA cm^−2^ is generally used to evaluate the bifunctional activity for catalyst. The Δ*E* of CMT@CNT achieves 0.678 V, smaller than that of ZIF-C (0.718 V), CMT (0.864 V), and Pt/C + Ir/C (0.763 V) (Fig. 4d), which also exceeds that of most bifunctional ORR/OER electrocatalyst reported in the studies (Table S4) [43–50].

### Zinc–Air Battery Performance

**Fig. 5:**
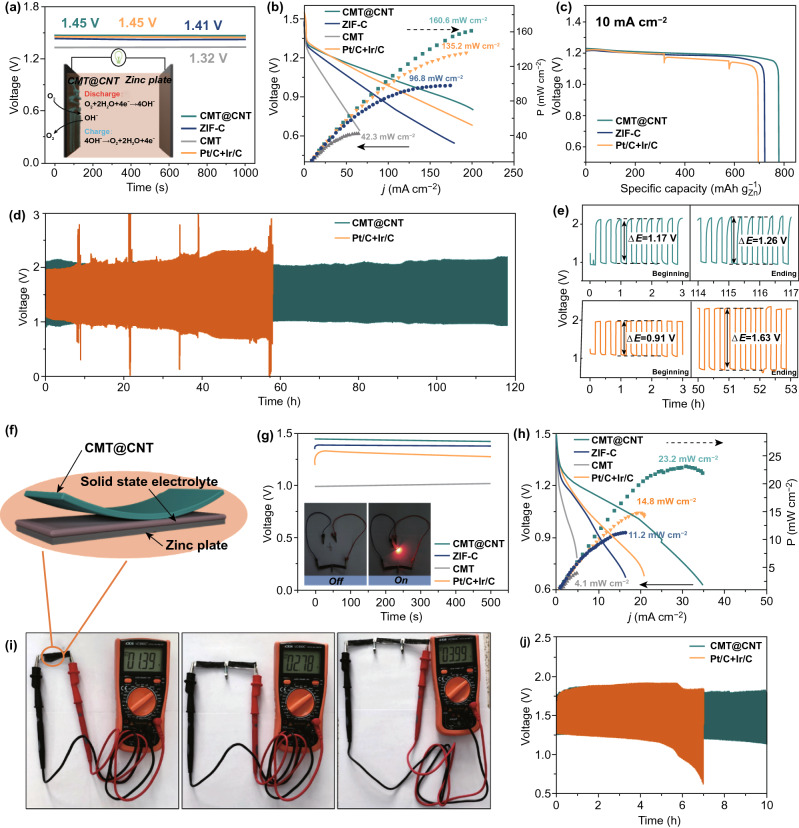
CMT@CNT, ZIF-C, CMT, and Pt/C + Ir/C-based ZABs and all-solid-state ZABs performance. **a** Open-circuit plots (inset: a schematic illustration of ZAB based on CMT@CNT).**b** Discharge polarization curves and the corresponding power density plots. **c** Specific capacity plots of CMT@CNT, ZIF-C, and Pt/C + Ir/C-based ZABs. **d,e** Discharge and charge curves of CMT@CNT and Pt/C + Ir/C-based ZABs. **f** A schematic illustration of all-solid-state ZAB based on CMT@CNT. **g** Open-circuit plots (inset: photograph of a red light-emitting diode powered by two CMT@CNT-based all-solid-state ZABs connected in series) and **h** discharge polarization curves and the corresponding power density plots. **i** Photograph of one, two, and three CMT@CNT-based all-solid-state ZABs in series displaying measured open-circuit voltage. **j** Discharge and charge curves of CMT@CNT-based and Pt/C + Ir/C-based all-solid-state ZABs

Inspired by the superior ORR and OER activities of CMT@CNT, a home-made ZAB has been constructed with CMT@CNT, zinc foil, and 6.0 M KOH/0.2 M zinc acetate as air cathode, anode, and electrolyte, respectively (Fig. 5a inset). For comparison, the ZABs with CMT, ZIF-C, and Pt/C + Ir/C (1:1) as air cathode were also made. Figure 5a displays that the open-circuit voltage of CMT@CNT-based ZAB (1.45 V) is comparable with that using Pt/C + Ir/C (1.45 V), which is higher than ZIF-C (1.41 V) and CMT-based ZABs (1.32 V). Moreover, the battery with CMT@CNT electrocatalyst shows a current density of 118.2 mA cm^−2^ at 1.0 V, much higher than that of Pt/C + Ir/C (93.9 mA cm^−2^), ZIF-C (63.8 mA cm^−2^), and CMT (27.6 mA cm^−2^) catalysts (Fig. 5b). The peak power density of CMT@CNT-based ZAB reaches 160.6 mW cm^−2^ at 198.8 mA cm^−2^, which is about 1.2, 1.7, and 3.8 times to that of Pt/C + Ir/C-based (135.2 mW cm^−2^ at 192.6 mA cm^−2^), ZIF-C-based (96.8 mW cm^−2^ at 164.5 mA cm^−2^), and CMT-based ZABs (42.3 mW cm^−2^ at 65.8 mA cm^−2^), respectively (Fig. 5b). Moreover, the CMT@CNT assembled ZAB exhibits a significant higher specific capacity and energy density (781.7 mAh g_Zn_^−1^ and 930.2 Wh kg^−1^) than Pt/C + Ir/C (696.4 mAh g_Zn_^−1^ and 814.8 Wh kg^−1^) and ZIF-C (723.5 mAh g_Zn_^−1^ and 853.7 Wh kg^−1^) at a constant current density of 10 mA cm^−2^ (Fig. 5c), which is one of the highest values among carbon-based electrocatalysts (Table S5) [43–46, 48–50], indicating its well application prospects. The cyclic stability and rechargeability of the constructed ZABs were further evaluated by galvanostatic discharge–charge cycling curve (Fig. 5d). The CMT@CNT-based ZAB shows an exceptionally superior stability for 117 h (351 cycles) at a current density of 20 mA cm^−2^, while Pt/C + Ir/C-based ZAB just maintained about 57 h (171 cycles). Briefly, an initial charge voltage of 2.14 V and discharge voltage of 0.97 V, as well as a voltage gap (Δ*E*, difference between charge voltage and discharge voltage) of 1.17 V was produced on the CMT@CNT-based ZAB. After galvanostatic discharge–charge cycling test, the charge voltage, discharge voltage, and voltage gap of the CMT@CNT-based ZAB are 2.21, 0.95, and 1.26 V (increased by 7.7%), respectively, endowing CMT@CNT a promising candidate for long-life ZAB (Fig. 5e). In contrary, the initial voltage gap and the last voltage gap of Pt/C + Ir/C-based ZAB are 0.91 and 1.63 V, respectively, with a high growth rate of 79.1%, demonstrating a serious decay in performance.

All-solid-state ZAB was also assembled with CMT@CNT, zinc foil, and alkaline polyvinyl alcohol (PVA) gel as air cathode, anode, and solid electrolyte, respectively, to investigate its application for flexible and portable devices (Fig. 5f) [51]. The CMT@CNT-based all-solid-state ZAB displays an open-circuit voltage of 1.44 V and a power density of 23.2 mW cm^−2^, higher than those of Pt/C + Ir/C (1.30 V, 14.8 mW cm^−2^), ZIF-C (1.38 V, 11.2 mW cm^−2^), and CMT (1.01 V, 4.1 mW cm^−2^) assembled all-solid-state ZABs (Fig. 5g, h). The open-circuit voltage of 1.39, 2.78, and 3.99 V was measured by multimeter for one, two, and three CMT@CNT-based all-solid-state ZABs connected in series (Fig. 5i). The stable performance under different folding angle indicates a high flexibility and promising potential in wearable electronics for CMT@CNT-based all-solid-state ZAB (Fig. S15). Moreover, it also exhibits a satisfactory cyclic stability and rechargeability with low Δ*E* after 10 h testing, much superior to Pt/C + Ir/C ZAB (Fig. 5j). Two CMT@CNT-based all-solid-state ZABs connected in series can power a red light-emitting diode without brightness change for 6 h, demonstrating the potential to satisfy the energy demands for actual application (Fig. 5g inset).

**Fig. 6:**
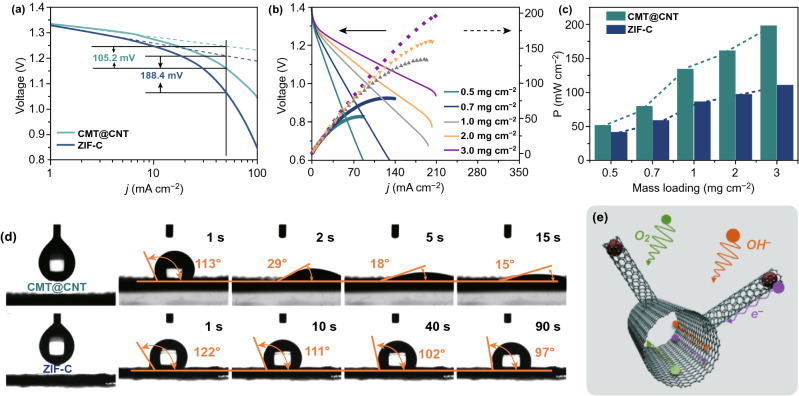
**a** Tafel slopes of discharge polarization curves for CMT@CNT and ZIF-C. **b** Discharge polarization curves and the corresponding power density plots of CMT@CNT-based ZAB at different catalyst mass loading. **c** Comparison of power density for CMT@CNT and ZIF-C. **d** Water contact of CMT@CNT and ZIF-C. **e** Schematic illustration of mass transfer process for CMT@CNT

It is known that the performance improvement of electrocatalysts is hard to maintain stable as the mass loading keep increasing, which is largely due to the limited electrolyte diffusion process and the resulted dead volume [29]. As we mentioned above, the unique hierarchical microtube@nanotube core–shell nanostructure should exert a significant effect on mass transport process and thus enhance electrochemical performance. In order to verify this state, the mass transport overpotential at a current density of 50 mA cm^−2^ for CMT@CNT and ZIF-C-based ZABs was extracted from the discharge polarization curves (Fig. 6a). The CMT@CNT electrocatalyst shows a much smaller overpotential of 105.2 mV compared to ZIF-C (188.4 mV), reflecting a significant boosted electrolyte diffusion process. Moreover, the ZAB performance under various mass loading was investigated for CMT@CNT and ZIF-C. It is exciting that the power density of CMT@CNT is gradually increasing from 51.7 to 196.6 mW cm^−2^ with a well linear trend when the mass loading increasing from 0.5 to 3.0 mg cm^−2^, indicating a mass loading independent behavior on this hierarchical microtube@nanotube core–shell nanostructure (Fig. 6b, c). However, the power density of ZIF-C grows much slower than that of CMT@CNT as the increasing of mass loading (Figs. 6c and S16).

It is reported that the hierarchical structured materials could promote hydrophilic wetting of electrolyte more effectively than bulk counterparts [33]. Thus, the structure advantages of CMT@CNT were further validated by contact angle measurements (Fig. 6d). The contact angle result reveals that the electrolyte drop could penetrate deep into the film with a contact angle from 113° to 15° in 15 s, demonstrating a higher surface hydrophilicity for CMT@CNT. On the contrary, the ZIF-C film shows a contact angle of 97° even after 90 s, demonstrating a higher electrolyte transport resistance. Based on the above results, the greatly enhanced oxygen electrocatalysis and ZAB performance of CMT@CNT over the traditional bulk materials can be ascribed to the following features (Fig. 6e): Firstly, the densely distributed CNT nanoarrays on this CMT render themselves with high intrinsic electrocatalytic activity and well dispersive active sites. Secondly, the CMT@CNT with hierarchical microtube@nanotube core–shell nanostructure can offer a high specific surface area and efficient transport path for electrolyte, which thus promote the contact between reactants and active sites and enhance the utilization of active sites (Figs. 2h, 3e, and 6d). Thirdly, the microtube skeleton could accelerate the electron transfer process due to the high content of graphitic carbon and graphitic-N (Figs. 2a, e, and 3f). Moreover, the one-dimensional tubular structure intertwined with each other could form a stable structure and thus deliver high electrochemical stability (Figs. 3g, 4c, 5d, j).

## Conclusions

In summary, we have developed a facile synthetic approach for preparing of hierarchical carbon microtube@nanotube core–shell nanostructure (denoted as CMT@CNT) and discussed the structure–activity relationship when employed in oxygen electrocatalysis and ZAB. The as-obtained CMT@CNT exhibits an extremely outstanding ORR and OER activity with a small potential gap of 0.678 V, which exceeds that of most reported carbon-based bifunctional electrocatalysts. The ZAB assembled by this CMT@CNT demonstrates a high power density of 160.6 mW cm^−2^ and specific capacity of 781.7 mAh g_Zn_^−1^ as well as long cycle stability over 117 h (351 cycles) at a current density of 20 mA cm^−2^. Moreover, CMT@CNT maintains its ZAB performance well even under high mass loading, which could meet the high demand of high power density and energy density for advanced electronic equipment. It is expected that the results will lend a new impetus to the rational design of more hierarchical structured electrocatalysts for clean and highly efficient energy conversion and storage technologies.

## Electronic supplementary material

Below is the link to the electronic supplementary material.

Supplementary material 1 (PDF 1119 kb)
