# Supplementary material for: Hierarchical Carbon Microtube@Nanotube Core–Shell Structure for High-Performance Oxygen Electrocatalysis and Zn–Air Battery
Source: Nanomicro Lett. 2020 Apr 20;12:97. doi: 10.1007/s40820-020-00435-z (PMC7770814; doi:10.1007/s40820-020-00435-z)
Supplement: Supplementary file 1 — Supplementary material 1 (PDF 1119 kb) [file 40820_2020_435_MOESM1_ESM.pdf]

Supporting Information for

Hierarchical Carbon Microtube@Nanotube Core-Shell Structure for High Performance Oxygen Electrocatalysis and Zn-air Battery

Wenfu Xie[1], Jianming Li[2], Yuke Song[1], Shijin Li[1], Jianbo Li[1], Mingfei Shao[1, *]

[1]State Key Laboratory of Chemical Resource Engineering, Beijing University of Chemical Technology, Beijing 100029, People's Republic of China

[2]Petroleum Geology Research and Laboratory Center, Research Institute of Petroleum Exploration & Development (RIPED), PetroChina, Beijing 100083, People's Republic of China

*Corresponding author. E-mail: shaomf@mail.buct.edu.cn (Mingfei Shao)

Supplementary Figures and Tables

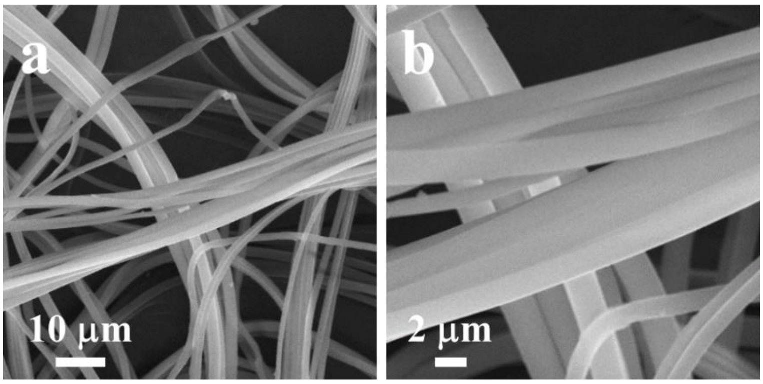

Fig. S1 SEM images of PP at different magnifications

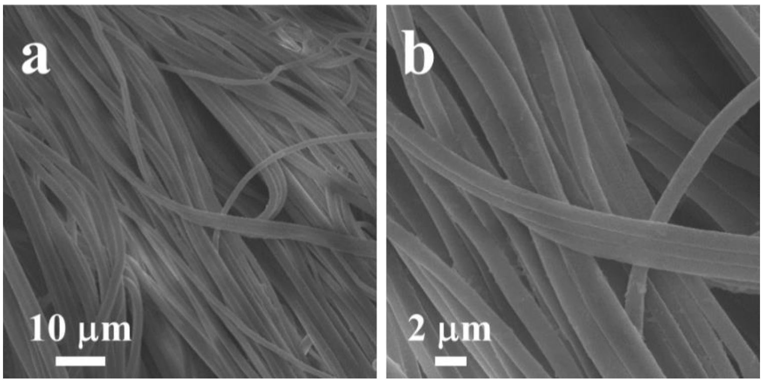

Fig. S2 SEM images of PP@PDA at different magnifications

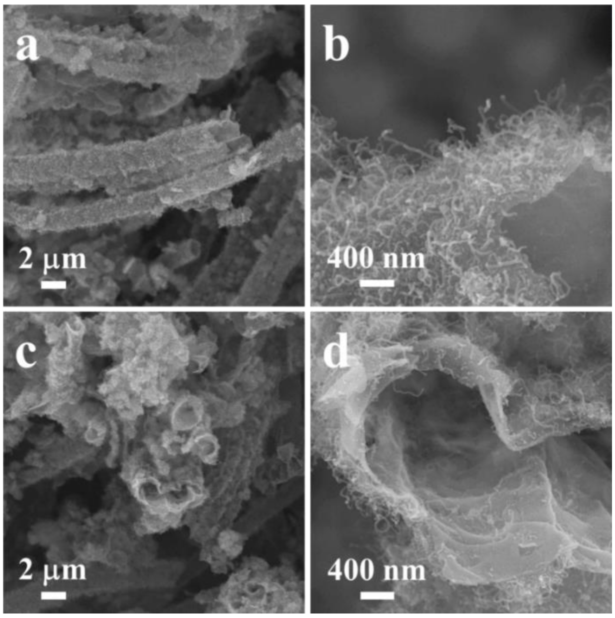

Fig. S3 SEM images of CMT@CNT at different magnifications

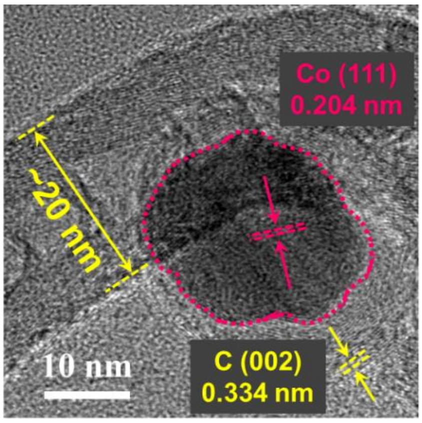

Fig. S4 HRTEM image of CMT@CNT

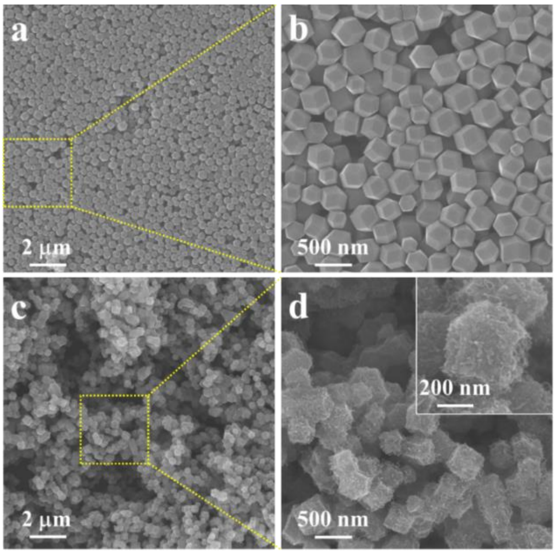

Fig. S5 SEM images of (a, b) ZIF-67 and (c, d) ZIF-C at different magnifications

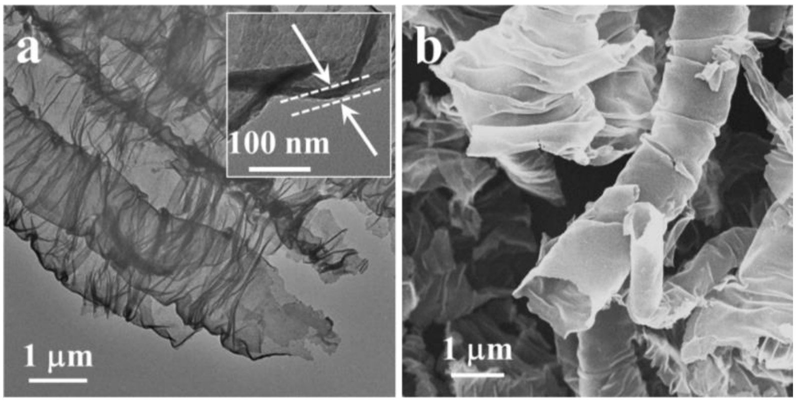

Fig. S6 (**a**) TEM and (**b**) SEM images of CMT

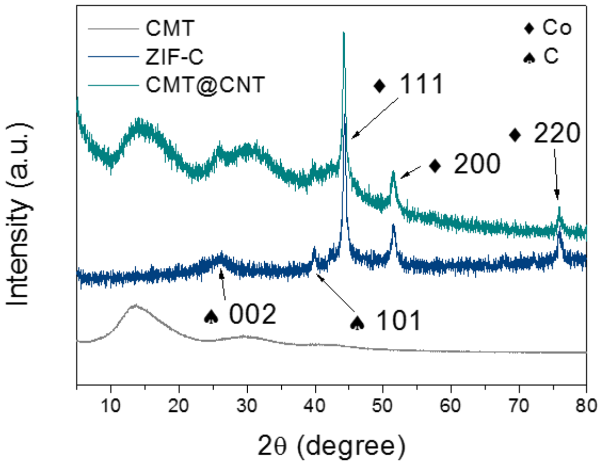

Fig. S7 XRD patterns of CMT@CNT, CMT and ZIF-C

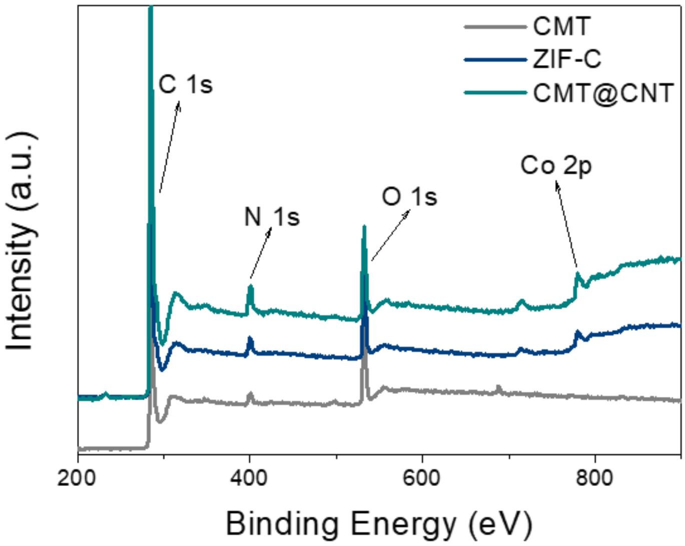

Fig. S8 XPS spectra of CMT@CNT, CMT and ZIF-C

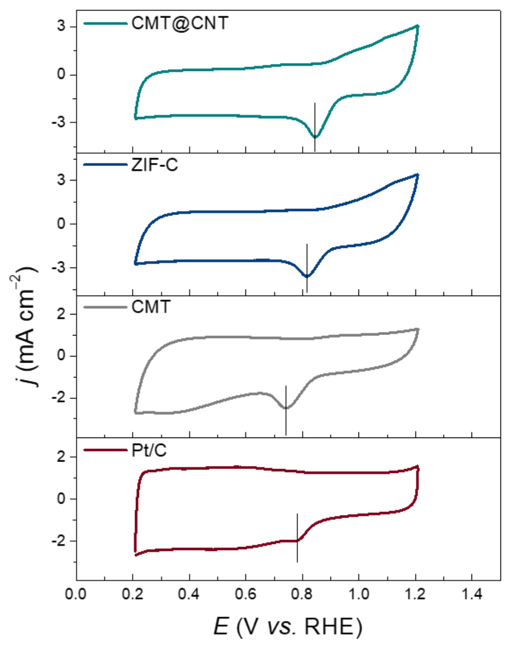

Fig. S9 CV curves of CMT@CNT, CMT, ZIF-C and Pt/C in O_2-saturated 0.1 M KOH

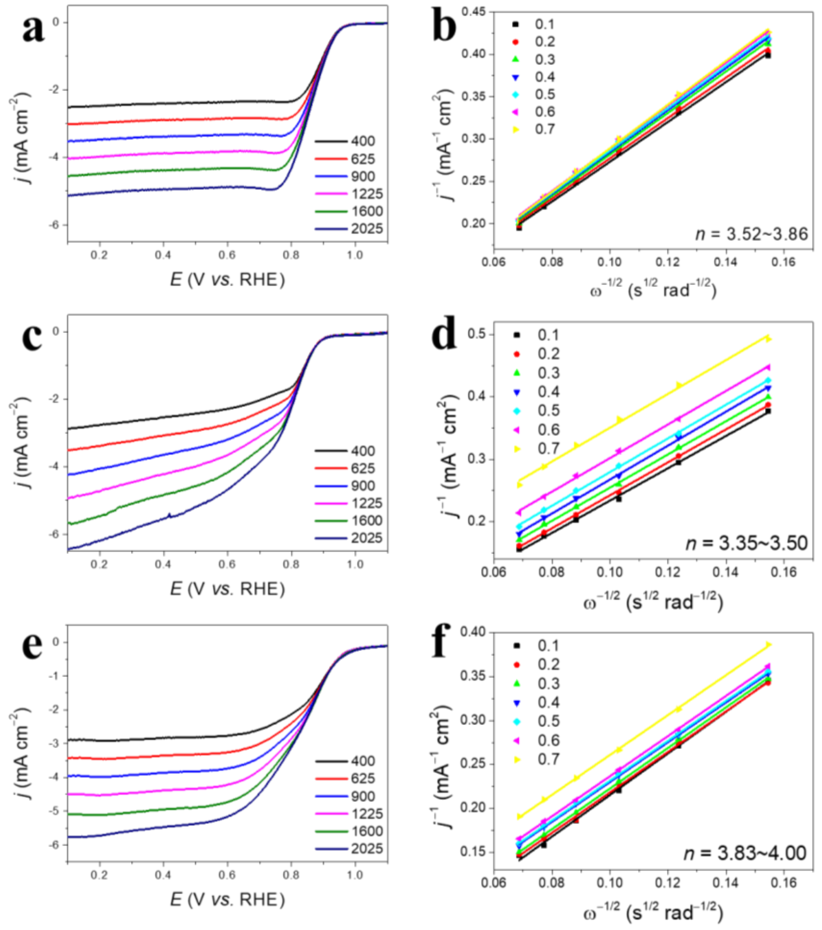

Fig. S10 LSV curves of (**a, b**) ZIF-C, (**c, d**) CMT, and (**e, f**) Pt/C for ORR at various rotation rates and the corresponding Kouteck–Levich plots at various potentials calculated from rotating disk electrode measurement data

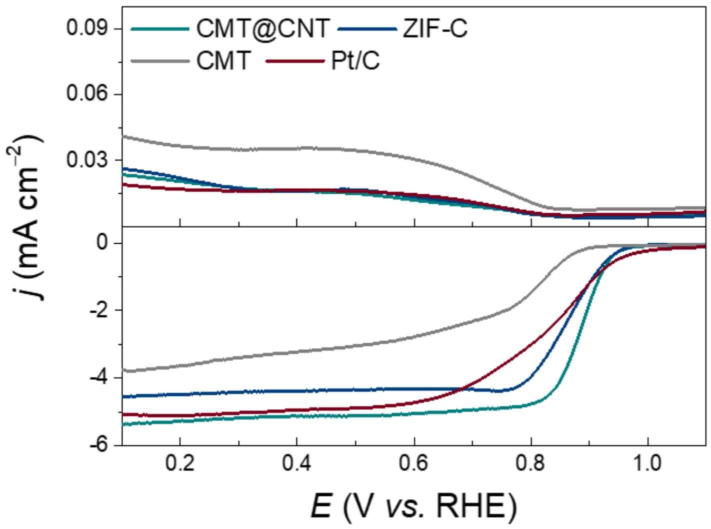

Fig. S11 RRDE measurements of CMT@CNT, CMT, ZIF-C, and Pt/C in O_2-saturated 0.1 M KOH

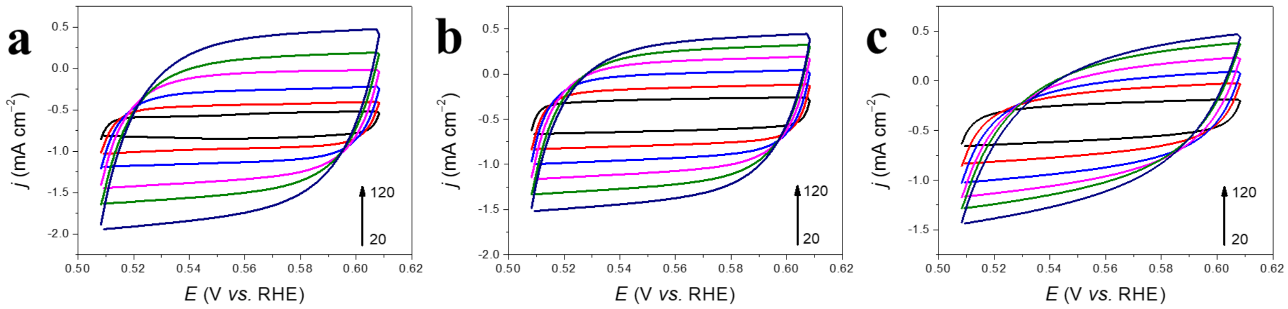

Fig. S12 CV curves of (**a**) CMT@CNT, (**b**) ZIF-C, and (**c**) CMT at various scan rates of 20, 40, 60, 80, 100, and 120 mV s^{-1}

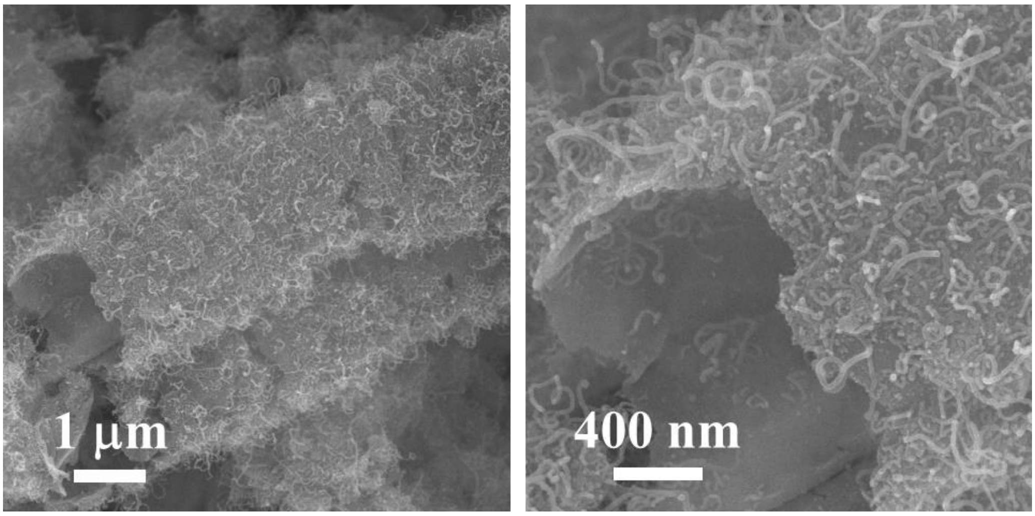

Fig. S13 SEM images of CMT@CNT after stability test with different magnification

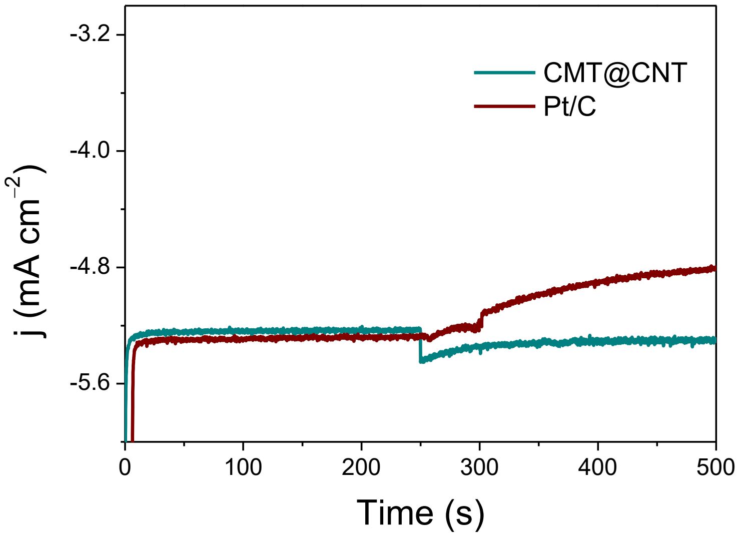

Fig. S14 *I-t* test of CMT@CNT and Pt/C with addition of methanol

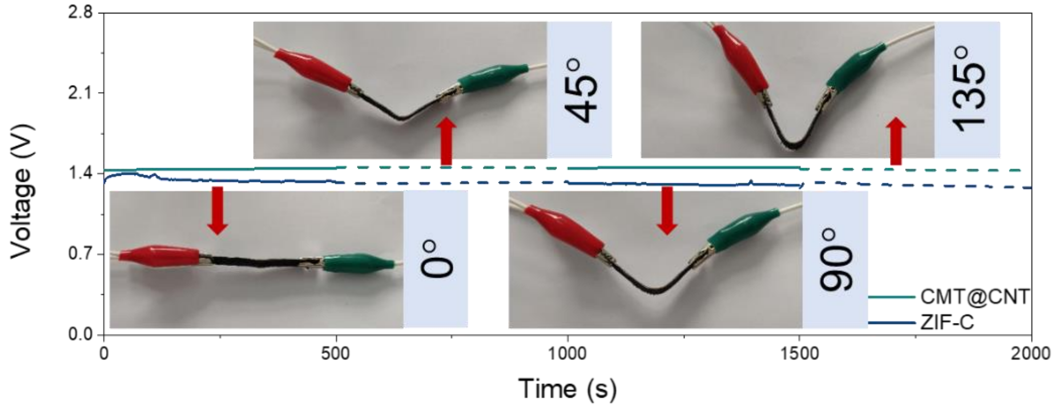

Fig. S15 Open-circuit plots of the all-solid-state ZABs based on CMT@CNT and ZIF-C bending at different angles

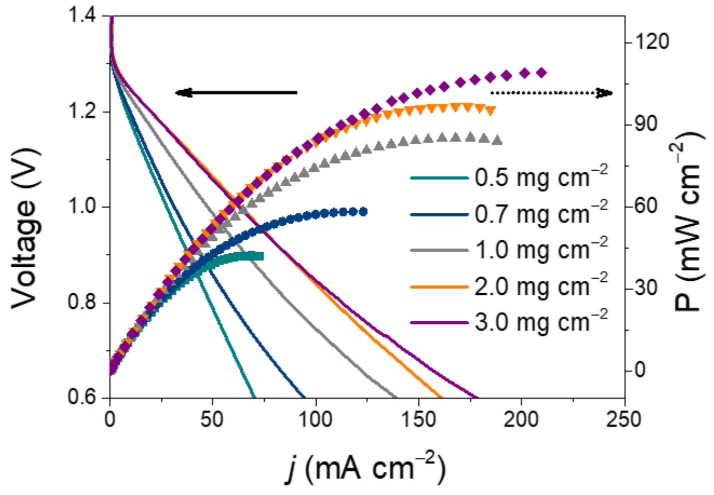

Fig. S16 Discharge polarization curves and the corresponding power density plots of ZIF-C-based ZAB at different catalyst mass loading

Table S1 Element contents measured by XPS

Sample	C (Atomic %)	N (Atomic %)	O (Atomic %)	Co (Atomic %)
CMT	86.66	7.53	4.81	0.00
ZIF-C	87.68	1.75	8.96	1.61
CMT@CNT	88.95	3.49	6.74	0.82

Table S2 Summary of N_2 sorption isotherm results

Sample	BET Surface Area (m^2 g^{-1})	Pore Volume (cm^3 g^{-1})	Pore Size (nm)
CMT	33.36	0.08	~31.56
ZIF-C	320.57	0.29	~7.59
CMT@CNT	354.27	0.51	~8.09

Table S3 Summary of electrochemical performance of CMT@CNT and references in this work

**Sample**	***j* for ORR (mA cm^{-2})**	**Half *E* (V_{RHE})**	**H_2O_2 (%)**	***n***	**η_{10} for OER (V)**	**Δ*E***
CMT	–3.92	0.75	10.66	3.78	384	0.864
ZIF-C	–4.61	0.86	4.12	3.91	348	0.718
CMT@CNT	–5.47	0.88	3.29	3.93	328	0.678
Pt/C	–5.43	0.82	3.39	3.93	-	0.763
Ir/C	-	-	-	-	353	

Table S4 Summary of electrochemical performance of CMT@CNT and the reported work

Sample	j for ORR (mA cm^{-2})	Half E (V_{RHE})	η_{10} for OER (V)	ΔE	Refs.
**CMT@CNT**	**–5.47**	**0.88**	**328**	**0.678**	**This work**
FeNC–S–Fe_xC/Fe	–5.66	0.887	320	0.680	S1
NB-CN	-	0.835	420	0.815	S2
NPCS-900	–5.45	0.830	420	0.820	S3
Co_3O_{4-x} HoNP-60	–5.82	0.834	313	0.740	S4
NiFe-LDH /Co,N-CNF	-	0.790	312	0.752	S5
$NiCo_2O_4$ /Co,N-CNTs	-	0.862	339	0.707	S6
NDGs-800	–5.60	0.850	450	0.830	S7
NCN-1000-5	–6.43	0.820	410	0.810	S8

Table S5 Summary of ZAB performance of CMT@CNT and the reported work

Sample	Open-circuit voltage (V)	Power density (mW cm^{-2})	Specific Capacity (mAh g_{Zn}^{-1})@j (mA cm^{-2})	Energy density (Wh kg_{Zn}^{-1})@j (mA cm^{-2})	Refs
**CMT@CNT**	**1.45**	**160.6**	**781.7@10**	**930.2@10**	**This work**
FeNC–S–Fe_xC/Fe	1.41	149.4	663@10	795@10	[S1]
NB-CN	1.4	320	-	-	[S2]
NPCS-900	-	79	625@20	656.25@20	[S3]
Co_3O_{4-x}	1.459	94.1	779.36@-	-	[S4]

HoNP-60					
$NiCo_2O_4$ /Co,N-CNTs	1.45	173.7	-	-	[S6]
NDGs-800	1.45	115.2	750.8@10	872.3@10	[S7]
NCN-1000-5	1.44	207	672@10	805@10	[S8]

Supplementary References

[S1]Y. Qiao, P. Yuan, Y. Hu, J. Zhang, S. Mu et al., Sulfuration of an Fe-N-C catalyst containing Fe_xC/Fe species to enhance the catalysis of oxygen reduction in acidic media and for use in flexible Zn-air batteries. Adv. Mater. **30**(46), e1804504 (2018). http://doi.org/10.1002/adma.201804504

[S2]Z.Y. Lu, J. Wang, S.F. Huang, Y.L. Hou, Y.G. Li et al., N, B-codoped defect-rich graphitic carbon nanocages as high performance multifunctional electrocatalysts. Nano Energy **42**, 334-340 (2017). http://doi.org/10.1016/j.nanoen.2017.11.004

[S3]S. Chen, L.L. Zhao, J.Z. Ma, Y.Q. Wang, L.M. Dai, J.T. Zhang, Edge-doping modulation of N, P-codoped porous carbon spheres for high-performance rechargeable Zn-air batteries. Nano Energy **60**, 536-544 (2019). http://doi.org/10.1016/j.nanoen.2019.03.084

[S4]D. Ji, L. Fan, L. Tao, Y. Sun, M. Li et al., The kirkendall effect for engineering oxygen vacancy of hollow Co_3O_4 nanoparticles toward high-performance portable zinc-air batteries. Angew. Chem. Int. Ed. **58**(39), 13840-13844 (2019). http://doi.org/10.1002/anie.201908736

[S5]Q. Wang, L. Shang, R. Shi, X. Zhang, Y.F. Zhao et al., NiFe layered double hydroxide nanoparticles on Co,N-codoped carbon nanoframes as efficient bifunctional catalysts for rechargeable zinc-air batteries. Adv. Energy Mater. **7**(21), 1700467 (2017). http://doi.org/10.1002/aenm.201700467

[S6]J. Li, S. Lu, H. Huang, D. Liu, Z. Zhuang, C. Zhong, ZIF-67 as continuous self-sacrifice template derived $NiCo_2O_4$/Co,N-CNTs nanocages as efficient bifunctional electrocatalysts for rechargeable Zn–air batteries. ACS Sustain. Chem. Eng. **6**(8), 10021-10029 (2018). http://doi.org/10.1021/acssuschemeng.8b01332

[S7]Q. Wang, Y. Ji, Y. Lei, Y. Wang, Y. Wang, Y. Li, S. Wang, Pyridinic-N-dominated doped defective graphene as a superior oxygen electrocatalyst for ultrahigh-energy-density Zn–air batteries. ACS Energy Lett. **3**(5), 1183-1191 (2018). http://doi.org/10.1021/acsenergylett.8b00303

[S8]H. Jiang, J.X. Gu, X.S. Zheng, M. Liu, X.Q. Qiu et al., Defect-rich and ultrathin N doped carbon nanosheets as advanced trifunctional metal-free electrocatalysts for the ORR, OER and HER. Energy Environ. Sci. **12**(1), 322-333 (2019). http://doi.org/10.1039/c8ee03276a
